# Leslie G. Ungerleider (1946–2020)

**DOI:** 10.1007/s00415-022-11421-3

**Published:** 2022-10-19

**Authors:** Stefano Sandrone

**Affiliations:** Department of Brain Sciences, Imperial College London, London, UK

**Fig. 1:**
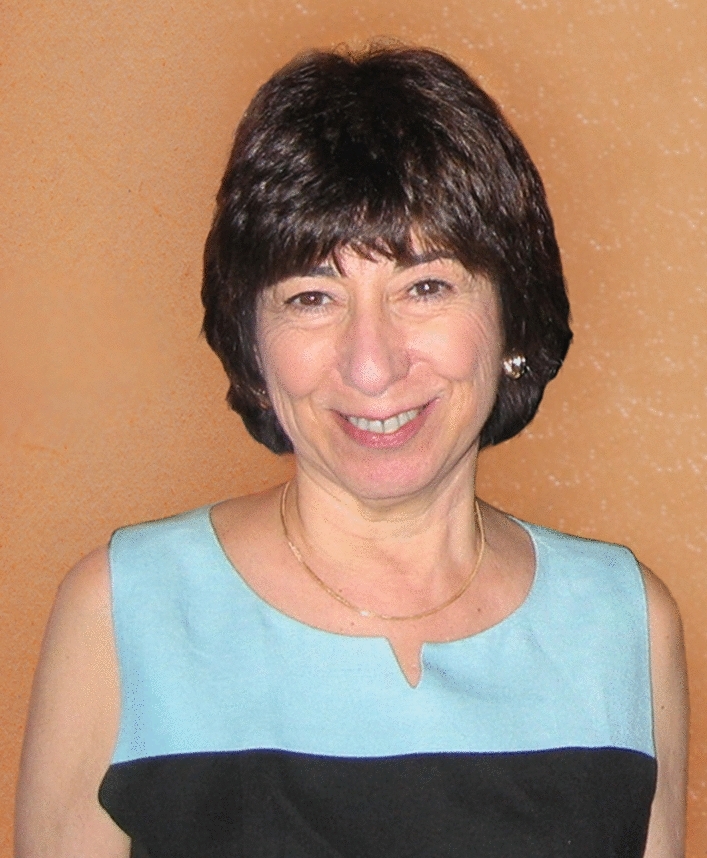
Portrait of Leslie G. Ungerleider. Credits: Michael Beauchamp

Leslie G. Ungerleider (Fig. 1) was born on 17 April 1946 in Queens, New York. She completed high school at age 16 and won the New York State’s Regents Scholarship to join Harper College (renamed State University of New York) in Binghamton, a small liberal arts college with a strong emphasis on experimental psychology [1]. She was awarded a B.A. *magna cum laude* in psychology in 1966 and joined New York University for her Ph.D. in experimental psychology [1]. In her words, the first year was a disaster, spent working with EEG and event-related potential scalp recording [1]. She even considered leaving the school but, after being reassigned to her mentor Edgar Coons, “life became idyllic” [1]. Together they published her first *Science* paper entitled ‘A behavioral measure of homosynaptic and heterosynaptic temporal summation in the self-stimulation system of rats’ in 1970, the same year she earned her Ph.D.

She next held a teaching appointment at the University of Oklahoma, a job chosen to follow her husband at the time, who had been offered a job at Oklahoma City. Two years later, the couple relocated to California, and Ungerleider started her first postdoctoral position at Stanford University. Although the plan was to collaborate with Leo Ganz on low-level vision in cats, she came across Karl Pribram, who was exploring high-level vision in non-human primates, and started working with him. They performed lesion experiments to investigate their effect on visual perception in macaque monkeys [2]. In 1974 she presented preliminary results at the annual meeting of the Society for Neuroscience. She was approached by Mortimer Mishkin, a scientific adversary of Pribram, as they had distinct views on the role of “pre-striate cortex […] of the inferior temporal cortex” and “their contributions to perception” [1]. As both Ungerleider and Mishkin were working on similar topics, albeit with different outcomes [1], Mishkin invited her to Bethesda, and in 1975 she started a second postdoctoral position at the National Institutes of Health (NIH), where she spent the rest of her career. In 1995 she established her own laboratory, becoming Chief of the Laboratory for Brain and Cognition at the National Institute of Mental Health, and in 2008 she became an NIH Distinguished Investigator.

Ungerleider and Mishkin discovered that animals with lesions of the inferior temporal cortex had difficulties identifying *what* an object was, and those with lesions of the posterior parietal cortex in locating *where* an object was [3, 4]. These two functionally dissociated cortical pathways, namely an occipitotemporal (ventral) pathway for the ‘what’ and an occipitoparietal (dorsal) pathway for the ‘where’ [5, 6], were later found in other sensory domains [3]; the light was shed on the neural circuitry with connectivity studies [2]. This concept was also linked to several perceptual functions and lesion syndromes, including agnosia, ataxia and neglect [3]. Throughout her career, Ungerleider explored the functional organization of the visual cortex in humans and non-human primates with neuroanatomical, neurophysiological, neuroimaging and behavioural methods [2, 7]. With the advent of neuroimaging, notably positron emission tomography (PET) and functional magnetic resonance imaging (fMRI), she made fundamental contributions to deciphering the neural basis of human cognition, perception and imagery, working memory and selective attention, emotional behaviour and decision-making [8]. She was an avid adopter of novel techniques [9, 10] and had a distinctive cross-disciplinary approach [6]. As a neuroanatomist, neurophysiologist and neuroimager [6, 9], she had a broad vision for the emergent field of cognitive neuroscience: she envisioned research areas before they became established subfields [6]. The breadth and depth of her scientific contributions, which often and quickly became textbook material [3], are simply incredible [9].

Ungerleider was kind yet blunt and honest [2], and rather intimidating at first [6]. Upon joining the NIH, she was somewhat uncomfortable in public speaking [6], but learnt from Patricia Goldman–Rakic to never turn down an invitation for a talk at a conference, a symposium or a workshop, and urged younger colleagues to follow that advice [10]. Over the years, she developed mastery in public speaking. Her slides and papers were meticulously prepared and displayed a clear logic and flow [2]. Attention to detail and a crystal-clear writing style were two of the aspects she passed on to her mentees; she often sat side-by-side with them to work on a draft, sentence by sentence [6], or practice talks before a conference, showing support and encouragement [2]. She nurtured a culture of curiosity and creativity, epitomised by her question: “What does it all mean?” when interpreting experimental data [3]. An inspiring role model, she also gave advice on negotiating start-up packages to female colleagues [2].

Ungerleider was a towering figure in the scientific community [3]. She was admitted to the National Academy of Sciences and the American Academy of Arts and Sciences in 2000 and the National Academy of Medicine in 2001, when she also won the Mika Salpeter Lifetime Achievement Award from the Society of Neuroscience. In 2004 she won the NIH Award for Mentoring, and in 2005 the George A. Miller Award from the Cognitive Neuroscience Society. In 2010 she was awarded the William James Award presented by the Association for Psychological Science. Jointly with Mortimer Mishkin, she won the University of Louisville Grawemeyer Award in Psychology in 2012. In 2011 she won the Golden Brain Award from the Minerva Foundation, in 2013 the Andrew Carnegie Prize, and in 2020 the Glass Brain Award of the Human Brain Mapping Organization. In 2021 the Society for Neuroscience awarded her the Patricia Goldman-Rakic Hall of Honor to highlight her excellent career and her work to promote and advance women in neuroscience.

Her last years were marred by personal and health tragedies [3]; nevertheless, her physical fragility revealed an even stronger resilience [2], and she remained fully engaged with science [2] until the end. She died on 11 December 2020 at the age of 74 years. Not only the ‘what’ and the ‘where’ of her neuroscientific journey, but even the ‘how’ behind it will remain a legacy for the next generations of neuroscientists.
